# Creating a Functional Biomimetic Cartilage Implant Using Hydrogels Based on Methacrylated Chondroitin Sulfate and Hyaluronic Acid

**DOI:** 10.3390/gels8070457

**Published:** 2022-07-21

**Authors:** Gerke H. Schuiringa, Marko Mihajlovic, Corrinus C. van Donkelaar, Tina Vermonden, Keita Ito

**Affiliations:** 1 Orthopaedic Biomechanics, Department of Biomedical Engineering, Eindhoven University of Technology, Gem-Z 1.106, P.O. Box 513, 5600 MB Eindhoven, The Netherlands; g.h.schuiringa@tue.nl (G.H.S.); mamih@dtu.dk (M.M.); k.ito@tue.nl (K.I.); 2 Department of Pharmaceutics, Utrecht Institute for Pharmaceutical Sciences (UIPS), Science for Life, Utrecht University, 3508 TB Utrecht, The Netherlands; t.vermonden@uu.nl

**Keywords:** cartilage tissue engineering, chondroitin sulfate methacrylate, hyaluronic acid methacrylate, hydrogel, HydroSpacer, spacer fabric

## Abstract

The load-bearing function of articular cartilage tissue contrasts with the poor load-bearing capacity of most soft hydrogels used for its regeneration. The present study explores whether a hydrogel based on the methacrylated natural polymers chondroitin sulfate (CSMA) and hyaluronic acid (HAMA), injected into warp-knitted spacer fabrics, could be used to create a biomimetic construct with cartilage-like mechanical properties. The swelling ratio of the combined CSMA/HAMA hydrogels in the first 20 days was higher for hydrogels with a higher CSMA concentration, and these hydrogels also degraded quicker, whereas those with a 1.33 wt% of HAMA were stable for more than 120 days. When confined by a polyamide 6 (PA6) spacer fabric, the volumetric swelling of the combined CSMA/HAMA gels (10 wt%, 6.5 × CSMA:HAMA ratio) was reduced by ~53%. Both the apparent peak and the equilibrium modulus significantly increased in the PA6-restricted constructs compared to the free-swelling hydrogels after 28 days of swelling, and no significant differences in the moduli and time constant compared to native bovine cartilage were observed. Moreover, the cell viability in the CSMA/HAMA PA6 constructs was comparable to that in gelatin–methacrylamide (GelMA) PA6 constructs at one day after polymerization. These results suggest that using a HydroSpacer construct with an extracellular matrix (ECM)-like biopolymer-based hydrogel is a promising approach for mimicking the load-bearing properties of native cartilage.

## 1. Introduction

Hydrogels are soft materials made of hydrophilic polymer networks that are able to absorb and retain water. In the past few decades, interest in these materials has increased significantly, especially in the fields of artificial implants, scaffolds, drug delivery, and wound healing [1,2,3,4,5,6]. Since they are able to mimic the extracellular matrix (ECM), hydrogels are particularly suitable for carrying and supporting cells [7,8,9,10], and are therefore applicable to tissue engineering and regenerative medicine. Naturally occurring glycosaminoglycans (GAGs), such as chondroitin sulfate (CS) and hyaluronic acid (HA), are very attractive materials for designing biomimetic hydrogels [11]. CS is a sulfated linear polysaccharide composed of glucuronic acid and *N*-acetylgalactosamine as its repeating disaccharide unit [12]. HA is also a linear polysaccharide, whose disaccharide repeating unit is composed of glucuronic acid and *N*-acetylglucosamine [13]. Both polymers are highly hydrophilic, negatively charged, and, therefore, characterized by water retention capacity and possessed of specific rheological, physiological, and biological properties. The resulting hydrogels have the potential to support encapsulated chondrocytes and are recommended for applications where chondrogenic potential is required [14,15,16,17]. However, the load-bearing function of articular cartilage tissue contrasts with the poor load-bearing capacity of most currently used and developed soft hydrogels [18,19].

The mechanical properties of native articular cartilage originate from the fact that the proteoglycans present in the tissue attract water due to an ion imbalance between the tissue and the surrounding fluid, and therefore induce tissue swelling. On the other hand, this swelling is restricted by the specific arcade-shaped, crosslinked collagen architecture present in the cartilage [20], inducing a Donnan osmotic pressure as the fixed charge density (FCD) remains high [21,22,23,24,25,26,27]. The more the swelling is prohibited, the higher the proteoglycan density and, therefore, the FCD are, and the better the load-bearing capacity is [28,29,30,31]. A drop to 2% of the original cartilage modulus was observed when the tissue was depleted of proteoglycans [32]. Moreover, the equilibrium response was found to be largely controlled by the osmotic pressure, as demonstrated by in vivo and in silico studies [27,33,34,35,36,37,38].

Both HA and CS belong to the family of GAGs that are naturally found in ECM and connective tissues, especially in articular cartilage. Therefore, these biopolymers are characterized by inherent cytocompatibility and bioactivity, which makes them suitable materials for clinical translation, e.g., for scaffolds in cartilage tissue engineering. Both methacrylated HA and CS, referred to as HAMA and CSMA, respectively, have individually been combined with other materials (both synthetic and natural) in order to achieve blends with improved properties, such as mechanical properties, swelling, biocompatibility, or application-oriented properties, as reviewed elsewhere [39]. However, CSMA and HAMA photocrosslinkable polymers have rarely been explored when combined alone, with no other additional materials. More often than not, CSMA and HAMA, when combined together, have also been joined by other biopolymers, such as collagen [40,41], gelatin-methacrylate (GelMA) [42], or alginate [43]. In most of these cases, the goal of such combinations was to intrinsically affect the final application, mainly by enhancing and regulating the chondrogenesis, and, in some cases, improving the mechanical properties (e.g., the stiffness) [42].

To improve the stiffness of hydrogels, fiber reinforcement has often been employed [44,45]. Another method is the use of warp-knitted spacer fabrics, consisting of a knitted top layer and a bottom layer connected by pile yarns, which have been shown to have a beneficial effect on the stiffness compared to plain hydrogels [46]. Such spacer fabrics restricted the swelling of negatively charged hydrogels (pHEMA-NaMA), thus generating a high osmotic pressure [47]. A significant positive correlation between the FCD of the hydrogel and the resulting stiffness of the construct was identified, and, with respect to the physiological cartilage-like FCD, the load-bearing properties were similar to those in cartilage, both in the loading phase and in the equilibrium phase [47].

**Scheme 1:**
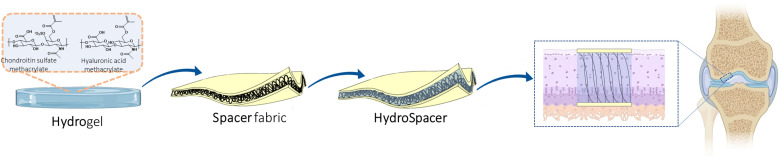
Hydrogel based on methacrylated chondroitin sulfate (CSMA) and hyaluronic acid (HAMA) is combined with polyamide 6 (PA 6) warp-knitted spacer fabric to form a HydroSpacer—a construct with spacer fabric material restricting hydrogel swelling and mimicking cartilage structural organization. Partially created with biorender.com.

Although the abovementioned study is very promising for the creation of implants for cartilage replacement with cartilage-mimetic load-bearing properties, it used very stable hydrogels that were non-regenerative and based on cytotoxic monomers. Thus, these gels cannot be used for clinical applications. The present study explores whether hydrogels based on the natural polymers CS and HA, injected into warp-knitted spacer fabrics (Scheme 1), could be used to create biomimetic constructs with cartilage-mimetic mechanical properties that would be stable over a longer time-period. The mechanical properties of these constructs, referred to as HydroSpacers, are characterized as a function of swelling achieved by varying the degree of hydrogel confinement in comparison to native articular cartilage.

## 2. Results and Discussion

### 2.1. Biopolymer Functionalization

**Figure 1:**
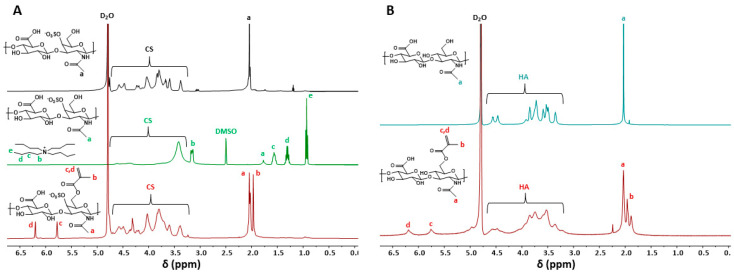
(**A**) H-NMR spectra of CS (top) in D_2_O, CS-TBA (middle) in DMSO d-6, and CSMA (bottom) in D_2_O. (**B**) H-NMR spectra of HA (top) in D_2_O and HAMA (bottom) in D_2_O.

During the preparation of the methacrylated polymers, the presence of proton peaks in the aliphatic region in the ^1^H-NMR spectrum (0.94, 1.32, 1.57, and 3.16 ppm) demonstrated the successful exchange of the sodium salt with TBA cations (Figure 1A). The following methacrylation reaction yielded CSMA polymer, as confirmed by the ^1^H-NMR spectrum, demonstrating the presence of methacrylate group proton peaks at 1.96, 5.77, and 6.20 ppm [48], while the peaks of the aliphatic protons disappeared, confirming the successful removal of the TBA ions (Figure 1A). The final CSMA polymer displayed a degree of methacrylation (DM) of ca. 23%, as determined by HPLC.

The formation of HAMA was confirmed by ^1^H-NMR with a DM of ca. 39%, where peaks corresponding to methacrylate protons were observed at 5.75 and 6.19 ppm (Figure 1B). All the detected peaks were in accordance with previously published results [48,49]. The methacrylation of both CS and HA was in line with the previously reported reactions, and both products were obtained as expected. It was important that the DM of HAMA exceeded 30%, as the crosslinking points deriving from HA methacrylate groups are considered to be responsible for the stability features of hydrogels. This notion is related to the higher stability of the ester methacrylate groups on HA polymers as compared to CSMA polymers [50].

### 2.2. Hydrogel Fabrication and Crosslinking Efficiency

**Figure 2:**
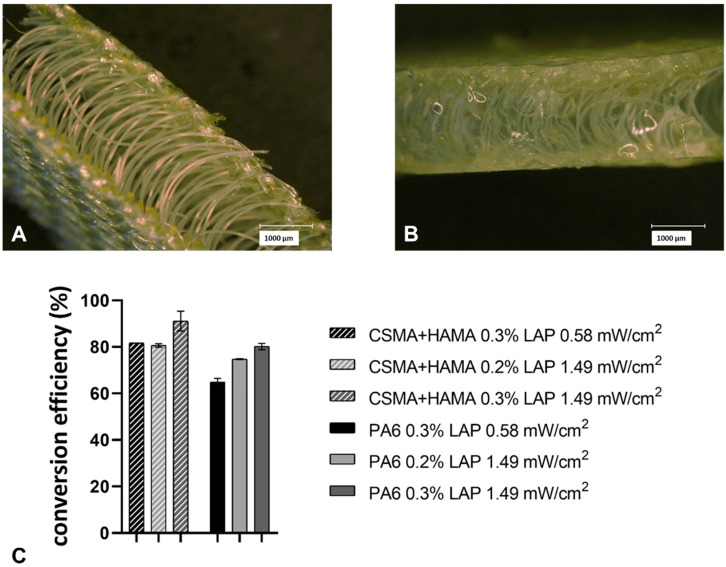
(**A**) Empty spacer fabric (PA6), scale bar 1000 µm. (**B**) HydroSpacer construct with CSMA/HAMA hydrogel formed with PA6, scale bar 1000 µm. (**C**) Methacrylate conversion efficiency of hydrogel samples with and without the addition of a PA6 spacer fabric (10 wt% polymers, CSMA to HAMA ratio 6.5), tested under different polymerization conditions and photo-initiator concentrations. Samples polymerized with a UV intensity of 0.58 mW/cm^2^ were exposed from one side for 15 min, whereas samples exposed to 1.49 mW/cm^2^ were bilateral polymerized for 7.5 min from each side. Samples were tested in duplicate, except for CSMA + HAMA 0.3% 0.58 mW/cm^2^ (*n* = 1). The data are presented as the mean ± SD.

The hydrogels were prepared via photopolymerization, and PA6 spacer fabric (Figure 2A) was used to confine the CSMA/HAMA hydrogels, yielding a HydroSpacer construct (Figure 2B). In order for the CSMA/HAMA hydrogels to be used as suitable cell carriers, the presence of unreacted methacrylate groups has to be limited [51]. The unreacted methacrylate groups, upon hydrolysis, produce methacrylic acid, which can cause cell toxicity due to its acidic character and reactivity to nucleophilic compounds [52,53,54]. Therefore, the crosslinking efficiency was optimized for the hydrogels and the hydrogels in spacer fabrics, where the PA6 influences the UV exposure (Table 1 and Figure 2).

With an LAP concentration of 0.3% and a bilateral exposure to UV at 1.49 mW/cm^2^, the hydrogels and HydroSpacers yielded the highest methacrylate conversions, at rates of 90 and 80%, respectively (Figure 2). The lower conversion can be explained by the spacer fabric blocking part of the light. Therefore, these conditions were chosen for the fabrication of the hydrogels to be used in further experiments.

### 2.3. Hydrogel Swelling Behavior

**Figure 3:**
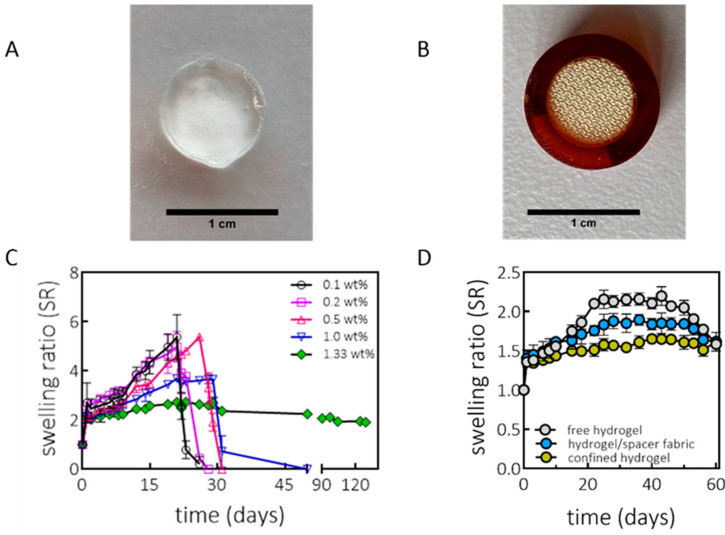
(**A**) CSMA/HAMA hydrogel disk after photopolymerization (10 wt% polymers, CSMA to HAMA ratio 6.5). (**B**) CSMA/HAMA hydrogel incorporated into spacer fabric and a cassette (confined hydrogel, 10 wt% polymers, CSMA to HAMA ratio 6.5). (**C**) Swelling ratio and stability in time of the CSMA/HAMA gels with different concentrations of the HAMA component (total polymer concentration was kept at 10 wt%). (**D**) Swelling profile of CSMA/HAMA hydrogels: a comparison between the free-swelling hydrogel, spacer fabric-filled hydrogel (semiconfined), and confined hydrogel (hydrogels were prepared with a 10 wt% polymer concentration, CSMA to HAMA ratio 6.5). All samples were measured in triplicate. Data are presented as the mean ± SD.

The swelling ratio of the CSMA/HAMA hydrogels (Figure 3A) in the first 20 days was higher for the hydrogels with the higher CSMA content and, therefore, higher FCD and lower crosslinking density, as determined by the DM of the polymers, and these hydrogels also degraded quicker after 20–30 days (Figure 3C). The swelling capacity of hydrogels is dependent on the crosslinking density, the latter also being indirectly related to the methacrylate ester stability. The hydrogels based on CSMA exhibited higher swelling potential and faster degradation rates compared to those based on HAMA (at comparable DM) due to the combined effects of the higher flexibility and hydrophilicity of CSMA compared to HAMA, rendering the microenvironment around the methacrylate esters in CSMA more hydrophilic and thus more sensitive to hydrolysis. This ester instability in CSMA eventually leads to a decreased crosslinking density, and thus causes more swelling [39,50,55,56,57,58]. The swelling profile observed in the formulations with a lower content of HAMA indicates a higher swelling capacity of the gels, which is most likely due to the more predominant CSMA component. This is also in line with previous research, where CSMA underwent a faster ester hydrolysis, which, in addition to a higher negative charge density (sulfate groups), resulted in water absorption and thus more swelling [48,59,60]. Consequently, different formulations produced different swelling profiles. The hydrogels containing 0.1–1 wt% of HAMA swelled to ~5.3 before fully degrading. The swelling profile of these gels indicates bulk degradation. The degradation of CSMA/HAMA hydrogels takes place through ester hydrolysis of the methacrylate groups [61]. These gels were stable for 30–58 days. With the incorporation of 1.33 wt% of HAMA, the hydrogels remained stable for over 120 days. This increased stability suggests that highly methacrylated HA at 1.33 wt% contributes to the stabilization of the hydrogels, as the methacrylate esters of HAMA are less sensitive to hydrolysis [62,63,64], and thus long-term stability can be achieved. The increased stability is also related to the limited swelling capacity observed in the formulation in question. In fact, CSMA/HAMA hydrogels with 1.33 wt% of HAMA displayed an SR of a maximum of ~2.5 within the first 30 days, after which it slowly decreased and equilibrated at ~1.9, demonstrating that, at 1.33 wt% HAMA, the network is sufficiently crosslinked to maintain a stable structure that is resistant to excessive swelling. This reduced swelling is due to the high DM of both polymers (23 and 39% for CSMA and HAMA, respectively) and in line with previously observed results in CSMA-based hydrogels [48,65]. This observation proved our hypothesis that making a hybrid hydrogel combining CSMA and HAMA could result in formulations with tunable swelling behaviors. It should be noted that CSMA-only-based gels degraded within ~3 weeks (15 wt%, pH 7.4), as reported previously [50], whereas those based on HAMA (10 wt%) could not be formulated due to the excessive solution viscosity.

Clearly, less swelling is directly related to higher stability, which is important for supporting chondrocytes over long periods. However, higher swelling capacity is important for creating osmotically induced pressurization of the scaffold, which is essential for mechanical load-bearing. These are contradicting requirements that were best met with the most stable formulation (1.33 wt% HAMA) that still displayed significant swelling, which will be investigated in more detail in the present work. The free gel reached the maximum swelling ratio of ~2.3 under free swelling after 21 days, until it started to reduce after ~50 days, continuing for the remaining 10 days of the study (Figure 3D). When confined by a spacer fabric (semi-confined), or by inserting the spacer fabric into a cassette (maximally confined, Figure 3D), the volumetric swelling of the gels was reduced by ~28% or ~53%, respectively. Even though the gels were confined axially and laterally in the latter case, there was still some swelling of the gels, as the cassette had a partially open structure. This experiment revealed that the swelling restriction did not influence the swelling significantly, except by reducing the maximum swelling in the period between days 20 and 40. Swelling then stayed constant, depending on the confinement, for another 20 days. However, interestingly, the stability was not affected by altering the swelling behavior, as between days 40 and 60, all differently confined gels had reduced swelling and each gel converged and equilibrated at ~1.6 of the swelling ratio at day 60.

The remaining question, then, is whether these confining conditions influence the mechanical properties of the materials by tuning the swelling behavior. Specifically, the stability of the hydrogels is mainly related to the swelling capacity (total water content) and also to the crosslinking density of the network [66].

### 2.4. Mechanical Characterization

**Figure 4:**
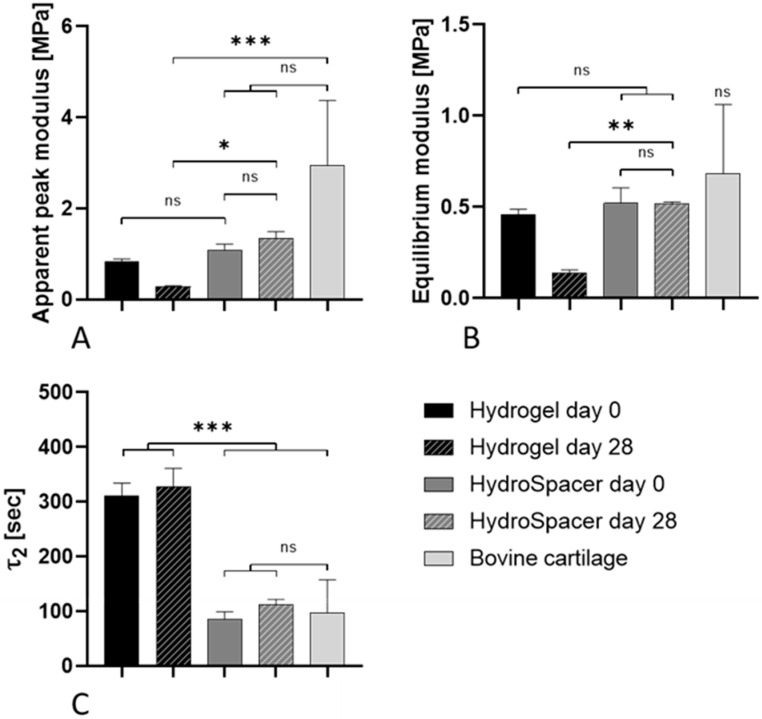
Mechanical properties of CSMA/HAMA hydrogels and HydroSpacers (*n* = 5) at day 0 and day 28, and of bovine cartilage. (**A**) Apparent peak modulus. (**B**) Equilibrium modulus. (**C**) Time constant τ_2_ in seconds. Data are presented as the mean ± SD. A Kruskal–Wallis test was performed using Prism GraphPad. The significance is indicated with *, ** or ***, with single signs referring to a *p*-value < 0.05, double signs referring to a *p*-value < 0.01 and triple signs referring to a *p*-value < 0.001.

Both the apparent peak and the equilibrium modulus significantly increased in the PA6-restricted constructs compared to the free-swelling hydrogels after 28 days of swelling. The more the gels swelled, the more the swelling potential was lost. If the swelling is restricted, the swelling potential is transferred into an osmotic pressure that strongly supports the load-bearing (Figure 4A,B). Moreover, after 28 days of swelling, the HydroSpacers showed no significant difference in the apparent peak, equilibrium modulus, and time constant τ_2_ compared to the native bovine cartilage, whereas the hydrogels alone showed significantly lower moduli and higher τ_2_ (*p* = 0.013, 0.008, and 0.032, respectively, for the apparent peak, equilibrium modulus, and τ_2_, Figure 4A–C). The addition of the spacer fabric to the hydrogel lead to a faster and increased relaxation of the construct, which was similarly observed in the native cartilage. This time-dependent behavior was also observed when a polyacrylamide-alginate hydrogel was introduced into a woven textile [67]. The effect of the addition of the spacer fabric on the time-dependent properties was already apparent at day 0. Possible explanations for this behavior could be, firstly, that PA6 absorbs water as well as the hydrogel [68], and, secondly, as PA6 is relatively inert, the addition of the spacer fabric might introduce an interface between the fibers and the hydrogel, thus creating channels around the fibers, both of which can influence the fluid flow while being compressed. There were no significant differences observed directly after polymerization between the hydrogels and the HydroSpacers, suggesting that the addition of the spacer fabric itself does not have a beneficial effect on the construct’s stiffness initially, but does have an effect after the swelling. The spacer fabric restricts the swelling and thereby, importantly, preserves the FCD, leading to an osmotic pressure which gives the HydroSpacers their load-bearing properties [47]. This observation is in line with the outcomes of numerical data showing that the load-bearing capacity of cartilage is highly dependent on the osmotic pressure [38], which is different from other reinforcement strategies using woven scaffolds, where the stiffness of the construct was dependent on the porosity and the pore size instead of the hydrogel used, as demonstrated in this study [67,69,70].

Although the mechanical testing technique employed in this study is not a pure form of confined compression or indentation, the values found in the literature for the aggregate modulus of both human and bovine cartilage are in the range of the values found in the current study [71,72,73,74]. This observation indicates that, when using hydrogels based on CSMA and HAMA, and restricting the swelling through a PA6 spacer fabric, load-bearing properties that are similar to those of native cartilage can be generated.

### 2.5. Cell Viability

**Figure 5:**
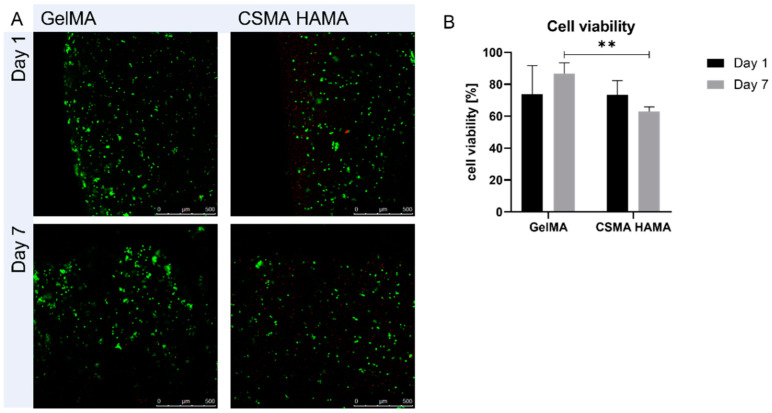
(**A**) Cell viability of gelatin–methacrylamide (GelMA) and CSMA/HAMA HydroSpacers after 1 and 7 days of culture. Viable cells are stained in green, dead cells in red. (**B**) Percentage of living cells embedded in the GelMA and CSMA/HAMA HydroSpacers (*n* = 5). The significance is indicated with **, with double signs referring to a *p*-value < 0.01.

On day 1, the cell death in the CSMA/HAMA HydroSpacers is concentrated at the outer edge, whereas in the GelMA HydroSpacers, it is diffused throughout (Figure 5A). However, no significant differences were observed in the overall cell viability between the CSMA/HAMA and GelMA HydroSpacers at day 1, reaching approximately 73% (Figure 5B). Cell viability increased over 7 days of culture in the GelMA constructs to 87%; however, this was not observed within the CSMA/HAMA HydroSpacers (63% viability), leading to a significant difference between the GelMA and CSMA/HAMA constructs at day 7. This difference might be caused by a stiffness-induced restricted cellular motility [75]. No effect of the PA6 spacer fabric itself was observed, as the cell viability was not hampered in the GelMA group, as compared to findings from the literature [76,77]. Nevertheless, the cell viability was sufficient to further investigate the CSMA/HAMA HydroSpacer as a potential strategy and as a regenerative application in cartilage tissue engineering. Moreover, the aim of this research was to demonstrate the initial cell viability, and an optimized cell culture system might lead to a higher cell viability which is more in line with previous research using chondroitin sulfate and hyaluronic acid-based hydrogels [78,79].

## 3. Conclusions

In this work, the naturally occurring polysaccharides CS and HA were successfully methacrylated and used for hydrogel fabrication. The swelling potential of the resulting hybrid CSMA/HAMA hydrogels was investigated as a function of the HAMA weight fraction. Hybrid hydrogels characterized by long-term stability (over 4 months) were prepared with a 1.33 wt% HAMA content (total polymer content 10 wt%). Moreover, the photopolymerization conditions of the hydrogels within the spacer fabric materials were optimized in order to ensure that the methacrylate conversion, and thus the crosslinking efficiency, would be as high as possible. A crosslinking efficiency of ~80% was achieved when the CSMA/HAMA hydrogels were fabricated within PA6 spacer fabric scaffolds, with no influence on cell viability compared to the GelMA HydroSpacers at one day after polymerization. The use of these PA6 spacer fabrics led to the restricted swelling of the CSMA/HAMA hydrogels, which resulted in the maintenance of the FCD within the CSMA/HAMA PA6 constructs. When using the osmotic pressure generated by the FCD of the CS and HA, the load-bearing properties were similar to those of native cartilage. The results demonstrated in this work suggest that the use of a HydroSpacer construct (with an ECM-like biopolymer-based hydrogel) is a promising regenerative approach for mimicking the load-bearing properties of native cartilage.

## 4. Materials and Methods

### 4.1. Materials

Sodium hyaluronate was purchased from Lifecore Biomedical (323 kDa as measured with GPC, Chaska, MN, USA). Chondroitin 4-sulfate sodium salt (bovine trachea) was obtained from Sigma-Aldrich (Zwijndrecht, the Netherlands). Lithium phenyl-2,4,6-trimethylbenzoylphosphinate was obtained from TCI Europe N.V (Zwijndrecht, the Netherlands). All organic solvents and reagents were purchased from Biosolve (Valkenswaard, the Netherlands) and Sigma-Aldrich (Zwijndrecht, the Netherlands), respectively, and were used without further purification. PA6 warp-knitted spacer fabrics were obtained from Karl Mayer Textilmaschinenfabrik GmbH (Obertshausen, Germany). Collagenase type II was purchased from Worthington Biochemical Corporation (Lakewood, NJ, USA). Hyaluronidase, fetal bovine serum (FBS, BCBV7611), and Calcein AM were purchased from Sigma-Aldrich. Dulbecco’s Modified Eagle Medium (DMEM, 41966-029, GibcoTM), penicillin/streptomycin (P/S, 15070063), and propidium iodide were purchased from Thermo Fisher Scientific (Landsmeer, the Netherlands/Waltham, MA, USA). Insulin/transferrin/selenium-plus (ITS+ premix, Corning) was obtained from VWR International B.V. (Amsterdam, the Netherlands).

### 4.2. Functionalization of the Biopolymers

The sodium salts of both the hyaluronic acid (HA) and chondroitin 4-sulfate (CS) were chemically modified to bear pending methacrylate moieties (Supporting information, Scheme S1). The methacrylation of HA was performed following a previously reported method [49]. Briefly, HA (4.8 g, 11.9 mmol) was dissolved in milliQ water (240 mL), and the solution was stirred overnight at 4 °C. Then, DMF (240 mL) was added, and the resulting mixture was placed in an ice bath. Methacrylic anhydride (5.5 mL, 36.9 mmol) was added dropwise at 0 °C (over 3.5 h), while continuously adjusting the pH between 8–9 (0.5 M NaOH). Next, the reaction mixture was supplemented with NaCl (0.5 M final concentration), followed by precipitation in cold ethanol. After filtration, the white precipitate was recovered, redissolved in milliQ water (550 mL), and dialyzed against water for 3 days (cutoff 14 kDa). The final product, corresponding to methacrylated HA (HAMA), was obtained after freeze-drying for 2 days as a white, cotton-like material (yield ~80%, defined as the ratio between the number of moles of the recovered HAMA and the starting HA, adjusting the molar mass of the HAMA according to the amount of the grafted methacrylate groups). For the methacrylation of CS, a previously published protocol was used [48]. Briefly, the sodium salt of CS was firstly converted into a more lipophilic tert-butyl-ammonium (TBA) salt (CS-TBA) through resin exchange (Dowex 50 × 8 w hydrogen form and tert-butyl-ammonium fluoride). The CS-TBA was then frozen and dialyzed (for 2 days against NaCl 150 mM aqueous solution, followed by 3 days against water, cutoff 14 kDa) and freeze-dried for 2 days. The dry CS-TBA (24.5 g, 31.1 mmol) was dissolved in anhydrous DMSO (935 mL), and the solution was stirred under N_2_ at 50 °C until the CS-TBA was fully dissolved. Then, 4-dimethylaminopyridne (DMAP) was added (4.5 g, 36.8 mmol), followed by glycidyl methacrylate (GMA) (5.1 mL, 37.3 mmol, feed ratio GMA:HA disaccharide 1.2), and it was allowed to stir at 50 °C for 65 h. The reaction mixture was diluted with milliQ water (water:DMSO ratio 1:1) and the pH was adjusted to 5.5 (0.2 M HCl). Finally, dialysis (for 3 days, cutoff 14 kDa) and freeze-drying for 2 days yielded the final product, chondroitin 4-sulfate methacrylate (CSMA), as a white-yellow fluffy solid (yield ~94%, defined as the ratio between the number of moles of the recovered CSMA and the starting CS, adjusting the molar mass of the CSMA according to the amount of the grafted methacrylate groups).

### 4.3. Determination of the Degree of Methacrylation with HPLC

The HAMA and CSMA polymers were accurately weighed (5 mg), placed in 2 mL of 0.02 M NaOH solution, and incubated overnight at 37 °C to allow for the basic hydrolysis of the methacrylate groups. Next, 1 mL of 2 M acetic acid was added to neutralize the solution. Methacrylic acid freed from the polymers was quantified with HPLC [80]. Specifically, the HPLC system was used (Alliance Waters), equipped with a UV-Vis detector (Dual Lambda absorbance, 210 nm) and Waters Sunfire C18 column. The eluent used had a ratio of 15:85 acetonitrile:milliQ water (v%), supplemented with 0.1% perchloric acid. The flow rate was set at 1 mL/min, and 10 µL of each sample was injected. The quantification was performed by means of a calibration curve of the methacrylic acid standards. The degree of methacrylation (DM) was defined as the number of methacrylate groups per the disaccharide units and expressed as a percentage.

### 4.4. ^1^H-NMR Spectroscopy

The NMR spectra of the functionalized biopolymers HAMA, CS-TBA, and CSMA were recorded on an Agilent 400-MR NMR spectrometer (Agilent Technologies, Santa Clara, CA, USA) in D_2_O. The chemical shifts were reported as δ in parts per million (ppm) and were calibrated against a residual solvent peak of D_2_O (δ = 4.79 ppm) or DMSO (δ = 2.50 ppm).

### 4.5. Hydrogel Fabrication

The CSMA and HAMA hydrogel disks were prepared by dissolving the polymers in PBS at the desired concentration (10 wt%). The resulting polymer solutions were supplemented with lithium phenyl-2,4,6-trimethylbenzoylphosphinate (LAP) photo-initiator (0.2 or 0.3 *w*/*v*% final concentration,) subsequently injected into a Teflon mold with cylindrical wells, and covered on both sides with quartz glass plates. The well dimensions for the free-swelling gels were 6 × 2 mm (diameter × height) and those for the confined gels were 8 × 3 mm (diameter × height). The crosslinking was achieved by UV-irradiating the samples for 15 min at distances of 3 or 5 cm from the light source for each side of the mold (UV lamp VL-4.LC, A. Hartenstein GmbH, intensity 0.58–1.49 mW/cm^2^, wavelength 365 nm). The HydroSpacers were prepared by inserting the warp-knitted PA6 spacer fabric (height: ~2.7 mm, diameter 8 mm) into the mold prior to filling the wells with the CSMA/HAMA hydrogel. The UV-polymerized HydroSpacers were transferred into cylindrical resin cassettes (8 × 3 mm, diameter × height). R05 resin (Envisiontec, Dearborn, MI, USA) was used to prevent the lateral swelling of the hydrogel. The spacer fabrics and HydroSpacers were visualized using a digital microscope (VHX-500F, Keyence Corporations, Osaka, Japan).

### 4.6. Crosslinking Efficiency Determination

Methacrylation conversion (crosslinking efficiency) after photopolymerization was determined with HPLC by measuring the amount of free methacrylic acid released after basic hydrolysis, corresponding to the methacrylate groups not reacted during photopolymerization (see Section 4.3). Instead of using free polymers, prepared hydrogels were freeze-dried and their weight was recorded (~60 mg).

### 4.7. Swelling

The hydrogel swelling capacity was determined by gravimetry. The unconfined hydrogel disks and HydroSpacers with and without cassettes were placed in pre-weighed vials. The initial weight (W_0_) was recorded, and the samples were incubated in 1 mL PBS (pH 7.4) at 37 °C. At designated time points, the hydrogel weight was determined (W_t_). The swelling ratio, defined as the ratio of W_t_/W_0_, was used to characterize the hydrogel swelling capacity. All samples were measured in triplicate.

### 4.8. Bovine Cartilage Harvesting

Full-thickness cartilage was harvested from bovine patellae (3–6 years old), which were collected after slaughter and stored at −20 °C. Prior to harvesting, the patellae were thawed at 4 °C for 24 h, immersed in PBS. Cartilage without visible fissures or roughening was isolated from the underlying bone of the distal-lateral quadrants of the patellae using a razor blade. Samples were immediately punched using an 8 mm-diameter biopsy punch (Curavet, Garbsen, Germany), positioned in the resin cassettes to ensure lateral confinement, and allowed to equilibrate in PBS at room temperature prior to mechanical characterization.

### 4.9. Mechanical Characterization

To determine the stiffness of the hydrogels, HydroSpacers, and cartilage plugs, such that the measurements were free of swelling or damage at the cut edges, an indentation test was performed using a 5 mm-diameter plane-ended indenter attached to a tensile tester (Model 42, MTS Criterion, Eden Prairie, MN, USA) equipped with a 50 N loadcell (LSB.503, MTS Systems Corp., Eden Prairie, MN, USA). The hydrogels and HydroSpacers were tested within the confined resin cassettes at day 0 and day 28. HydroSpacers were already kept in these cassettes. After 28 days of swelling in PBS at 37 °C, the free-swelling hydrogels were cut to fit into the resin cassettes using an 8 mm-diameter biopsy punch (Curavet, Garbsen, Germany).

A stress relaxation test was performed by applying 15% strain, relative to the equilibrium height after swelling, with a strain rate of 15%/sec, in PBS. The strain was held constant for 600 s and the stress relaxation was measured at a frequency of 10 Hz. The apparent peak, equilibrium moduli, and time constant τ_2_ were calculated from the relaxation curve using curve fitting (Equation (1), using Matlab (Mathworks Inc., Natick, MA, USA):

(1) $$\sigma_{t}=a+b^{\left(-t/\tau_{1}\right)}+c^{\left(-t/\tau_{2}\right)}$$

where *t* is the test time in seconds and *a, b* and *c* are constants.

### 4.10. Cell Viability

The chondrocytes were isolated from bovine metacarpal joints (aged 8–12 months, slaughterhouse material) using a previously described enzymatic digestion method [49]. After harvesting, the cells were suspended in either 10 % (*w/v*) GelMA or 10 wt% CSMA/HAMA at a concentration of 10 × 10^6^ cells/mL, injected into the PA6 spacer fabrics and polymerized as previously described. The HydroSpacers were cultured in DMEM, 41966-029, supplemented with 1% ITS+ premix and 1% P/S for up to 7 days. The cell viability was assessed using a live/dead assay. The samples were cut in half and stained, for living cells using Calcein AM (2 µM) and for dead cells using propidium iodide (1.5 µM), in PBS for 60 min at 37 °C, and visualized using confocal microscopy (Leica TCS SP5X, Wetzlar, Germany) after 1 day and 7 days of culture.

### 4.11. Statistics

The data are presented as the mean ± standard deviation. A Shapiro–Wilk test was performed to check for normality. If the samples were normally distributed, a two-way ANOVA test with Tukey’s multiple comparison post hoc testing was performed; otherwise, a Kruskal–Wallis test with a Dunn’s multiple comparison test was performed. All analyses were performed using Prism GraphPad. A *p*-value < 0.05 threshold was used to indicate significant differences between the groups (* *p* < 0.05, ** *p* < 0.01, *** *p* < 0.001).

## Figures and Tables

**Table 1:** Conditions tested to optimize the conversion efficiency of the methacrylate groups upon photopolymerization.

Sample ^a^	Spacer Fabric	LAP (*w/v*%)	Exposure Duration (min)	UV Exposure (Direction)	UV Intensity (mW/cm^2^)
CSMA/HAMA	No	0.3	15	One side	0.58
CSMA/HAMA	No	0.2	15 (7.5 each side)	Both sides	1.49
CSMA/HAMA	No	0.3	15 (7.5 each side)	Both sides	1.49
CSMA/HAMA/PA6	Yes	0.3	15	One side	0.58
CSMA/HAMA/PA6	Yes	0.2	15	Both sides	1.49
CMSA/HAMA/PA6	Yes	0.3	15 (7.5 each side)	Both sides	1.49

^a^ All hydrogel samples were prepared with 10 wt% polymer concentration (HAMA 1.33 wt%).

## Data Availability

All data generated or analyzed during this study are included in the published article and Supplementary Materials.

## Supplementary Materials

The following supporting information can be downloaded at: www.mdpi.com/article/10.3390/gels8070457/s1, Scheme S1: Synthesis route for HAMA and CSMA polymers.
