# Supplementary material for: Creating a Functional Biomimetic Cartilage Implant Using Hydrogels Based on Methacrylated Chondroitin Sulfate and Hyaluronic Acid
Source: Gels. 2022 Jul 21;8(7):457. doi: 10.3390/gels8070457 (PMC9315485; doi:10.3390/gels8070457)
Supplement: Supplementary file 1 [file gels-08-00457-s001.zip › gels-1795547-supplementary.pdf]

Supporting information

In order to prepare hydrogels from CS and HA biopolymers, they first have to be functionalized with methacrylate groups. CS was methacrylated following a previously described method where it was first converted to a more lipophilic form (CS-TBA) and subsequently was methacrylated in organic solvent (DMSO), in the presence of DMAP and GMA (Scheme 1 bottom)[1].

Scheme S1. Synthesis route for HAMA (top) and CSMA (bottom) polymers.

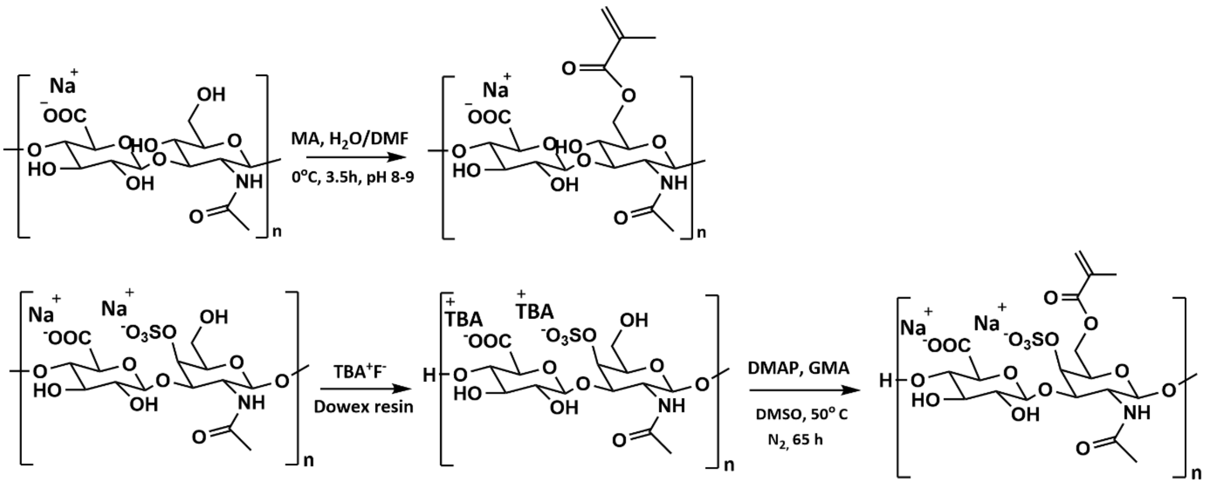

(1) Abbadessa, A.; Blokzijl, M. M.; Mouser, V. H. M.; Marica, P.; Malda, J.; Hennink, W. E.; Vermonden, T. A Thermo-Responsive and Photo-Polymerizable Chondroitin Sulfate-Based Hydrogel for 3D Printing Applications. *Carbohydr. Polym.* **2016**, *149*, 163–174. https://doi.org/10.1016/j.carbpol.2016.04.080.
